# Using NIST Crystal Data Within Siemens Software for Four-Circle and SMART CCD Diffractometers

**DOI:** 10.6028/jres.101.030

**Published:** 1996

**Authors:** Susan K. Byram, Charles F. Campana, James Fait, Robert A. Sparks

**Affiliations:** Analytical Instrumentation Group, Siemens Energy and Automation, 6300 Enterprise Lane, Madison, WI 53719-1173

**Keywords:** CCD diffractometer, crystallographic databases, database search, four-circle diffractometers, NIST Crystal Data, single crystal structures

## Abstract

NIST Crystal Data developed at The National Institute for Standards and Technology has been incorporated with Siemens single crystal software for data collection on four-circle and two-dimensional CCD diffractometers. Why this database is useful in the process of single crystal structure determination, and how the database is searched, are described. Ideas for future access to this and other databases are presented.

## 1. Introduction

As a commercial vendor of single crystal x-ray diffractometers, Siemens^1^ both produces data which goes into crystallographic databases, and searches these databases during the process of single-crystal structure determination. This paper describes how NIST Crystal Data is incorporated with the graphical user interfaces of the XSCANS software for single crystal diffractometers, and of the SMART software for two-dimensional CCD diffractometers. Reasons for choosing this particular database and ideas for future access to this and other crystallographic databases are described.

## 2. Primary Application of NIST Crystal Data Search

Siemens small-molecule diffractometer users are also users of the NIST crystal data identification file. The diffractometer users are researchers studying new chemicals, minerals and pharmaceuticals. In decreasing order of frequency, their fields are chemistry (inorganic, organometallic, and organic), crystallography, mineralogy, and materials science. In North America, their affiliations are 75% academic, 15% industrial, and 10% government. The primary application in searching NIST Crystal Data is compound identification. Specifically, it is to compare the unit cell of a new single crystal specimen being studied on the diffractometer to previously studied unit cells in the database. It is important to note that this search can be done before spending the time to collect the single-crystal data itself. Thus, the primary reason to search NIST Crystal Data is to maximize productivity by not re-collecting data on a known compound.

### 2.1 How Often Known Structures are Redetermined

Even highly experienced researchers may find themselves redetermining a known compound as the number of new single crystal structures grows far beyond human ability to remember each one. There are 197 500 entries with lattice parameters in the 1994 Version of NIST crystal data, incorporating entries on all classes of crystalline materials such as inorganics, minerals, metals, intermetallics, organics, and organometallics. Cases of crystal-structure redetermination of common starting materials which were recrystallized, and redeterminations of compounds previously reported in lesser known or foreign-language journals, have been reported to us from time to time. To try to attach a quantitative figure for how often these redeterminations happen, the results of the Pittsburgh Summer School in crystallography were surveyed from 1992 through 1995. This school provides a 10 day course of intensive lectures and hands-on data collection for approximately 30 students each summer. Each student is asked to bring a crystal of an unknown compound on which to collect data and solve the structure. Each year, one or two of these students has unexpectedly found a match for the supposedly unknown specimen. Complete results are given in Table 1. If the NIST Crystal Data search is done prior to data collection, the student has time to study a different unknown compound. As more and more classes incorporate complete structure determinations as projects, the NIST Crystal Data search will become more important.

## 3. How the NIST Crystal Data Search is Implemented

The algorithms to search NIST Crystal Data have been written at Siemens, since it was necessary to embed them in the graphical user interfaces which control the serial four-circle or two-dimensional CCD diffractometers. Both types of instruments are controlled by a PC, with a CD-ROM attached which contains the NIST Crystal Data Identification File obtained through the International Center for Diffraction Data [1]. An index file for very fast search is created locally on the PC hard disk by a Siemens utility program when the file is first installed and when it is updated annually. A simple point-and-click menu initiates the NIST Crystal Data search, utilizing the unit-cell information already determined by the diffractometer. When a match for the unit cell is found within user-chosen tolerances, the CD-ROM is accessed for detailed information on the known unit-cell. This information includes the known unit cell parameters *a*, *b*, *c*, *α*, *β*, *γ*, and cell volume; compound name and formula; literature reference; and some descriptive information. The information is displayed and stored for future access.

The original algorithms incorporate many ideas derived from discussions with Alan Mighell, Vicky Karen, and colleagues at NIST as far back as 1977 when NIST was the National Bureau of Standards [2,3,4]. The algorithms were updated after the 1992 American Crystallographic Association meeting, where Rodgers and LePage [5] discussed searching NIST Crystal Data when large uncertainties are present. Cell-comparison techniques discussed by Andrews et al. [6] have also been considered. Algorithms are designed to ensure that no known unit cells are missed in the search. The output may sometimes present numerous candidates for a match, but this can be screened readily by the researcher and is not considered problematic since the search is done only once per new crystal studied.

### 3.1 Four-Circle Serial Diffractometers

**Fig. 1:**
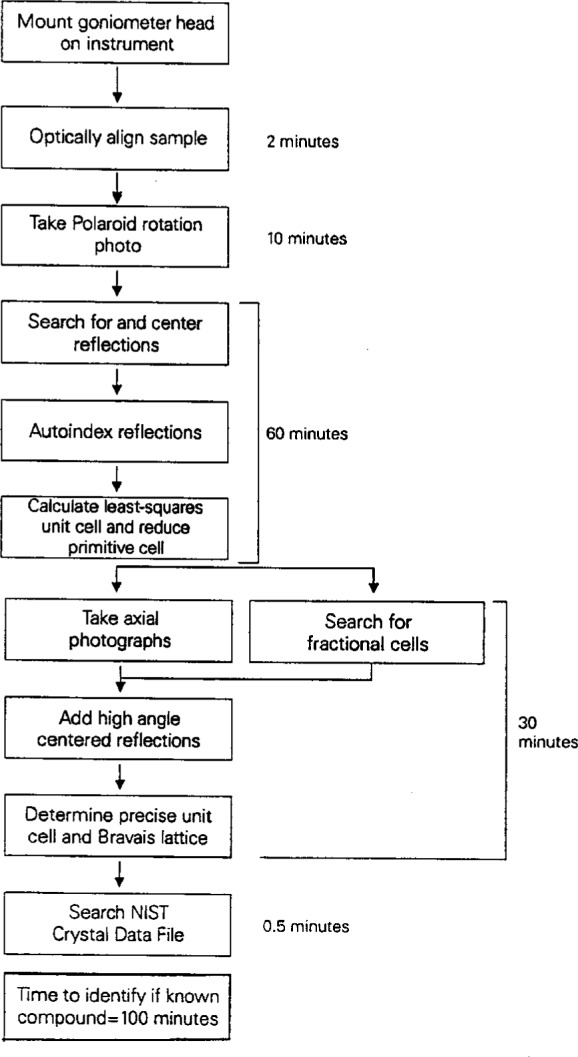
Four-circle diffractometer search time.

**Fig. 2:**
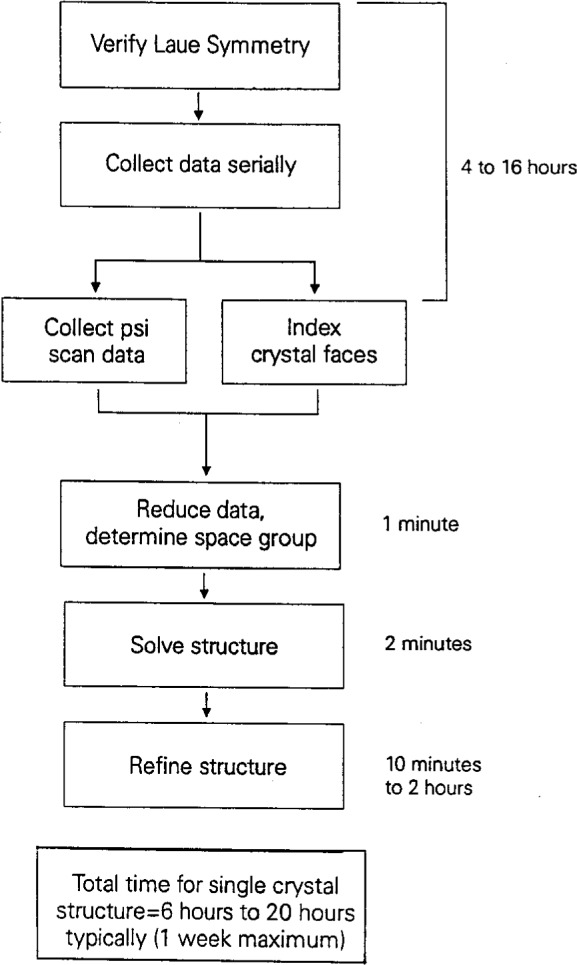
Time to collect data on a four-circle diffractometer.

When the crystal data are to be collected on a serial four-circle diffractometer, the average time to proceed from mounting the unknown crystal on the diffractometer to determining the precise unit cell and Bravais lattice is approximately 1 1/2 hours, as shown in Fig. 1. This assumes no human intervention between initial screening for the suitability of the crystal and initiating the NIST Crystal Data search. This point, prior to data collection, is the best time to search the NIST Crystal Data to see if the compound on the diffractometer has been studied previously, since a further 6 h to 20 h typically will be needed to collect the single-crystal data and to solve the structure, as shown in Fig. 2. If the unit cell is very large or the crystal weakly diffracting, this time could extend to many days of data collection. Thus the human and instrument time productivity is much increased if this time is not repeated.

### 3.2 Two-Dimensional CCD Diffractometer

**Fig. 3:**
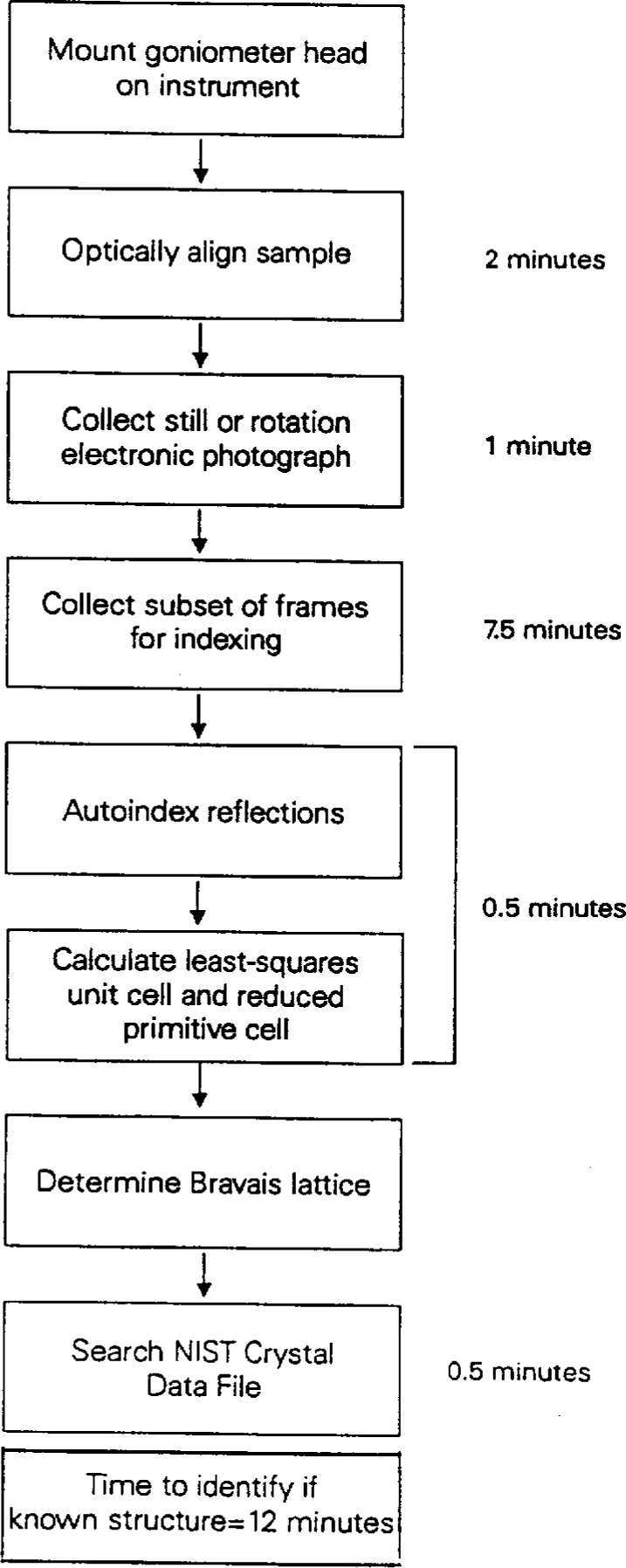
CCD diffractometer search time.

**Fig. 4:**
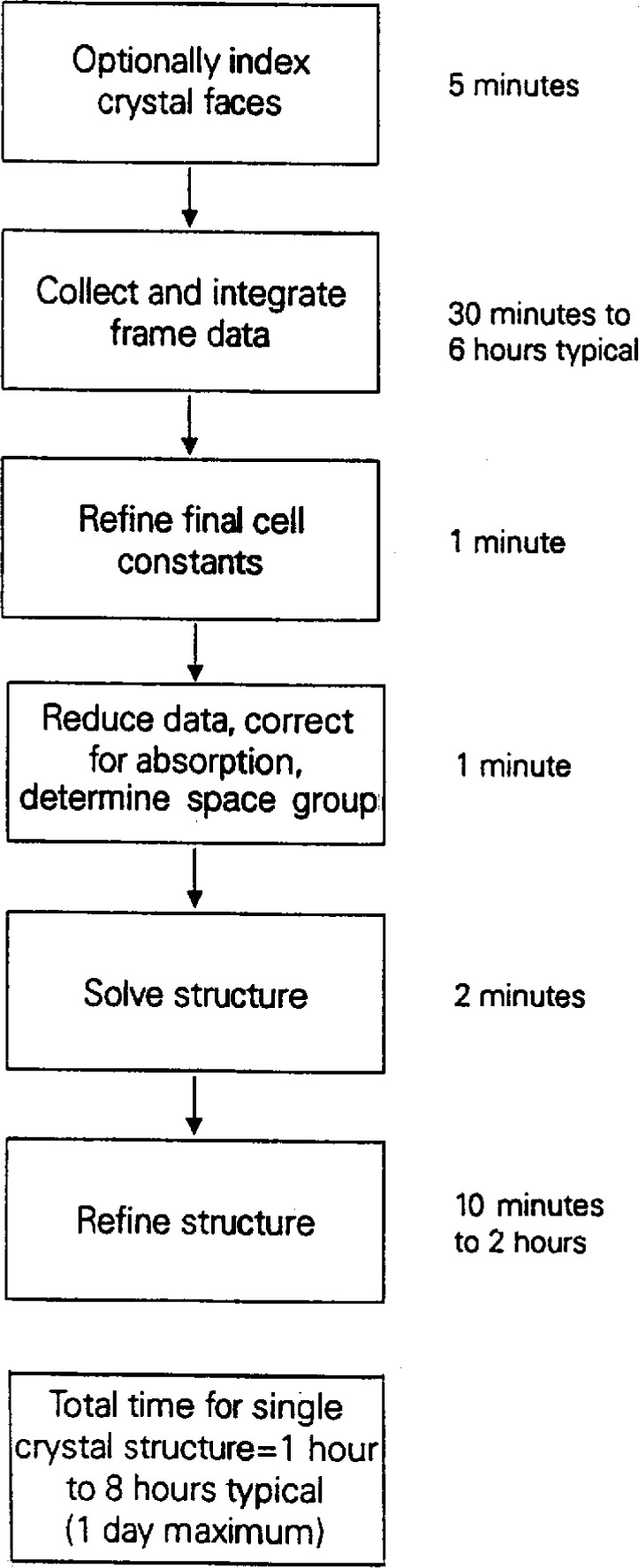
Time to collect data on a CCD diffractometer.

Siemens has recently introduced a new type of diffractometer for collecting data on single crystals. This is called SMART (Siemens Molecular Analysis Research Tool) and incorporates a novel two-dimensional CCD detector capable of collecting many reflections simultaneously [7]. With this new instrument, the average time to proceed from mounting the unknown crystal on the CCD diffractometer to determining the precise unit cell and Bravais lattice is only 12 min, as shown in Fig. 3. This short time leads us to believe that the SMART system, coupled with a NIST Crystal Data search, could be used to identify unknown single crystals in much the same way powder samples are identified using a powder diffractometer today. The initial reason for searching the NIST Crystal Data, to avoid re-collecting data on a known compound, is still important. From Fig. 4, we see that a further 1 h to 8 h to collect the data and solve the structure is required. Many researchers choose a CCD diffractometer system for crystals which are very weakly diffracting or very small, for example 10 μm on a side. For such crystals data collection may take up to a day.

### 3.3 Examples of NIST Crystal Data Search

The search is initiated using precise unit-cell parameters, determined on the diffractometer immediately prior to the search, or typed into the search menu from previously determined data. Parameters used by the sarch are: unit-cell axial lengths *a*, *b*, *c*; axial angles *α*, *β*, *γ*; tolerance for the match as a fraction; lattice centering type (such as P for primitive); whether to search the organic or inorganic file or both; an output file name to store results; and whether to display short or verbose output.

Typically no hits will be shown, meaning no match is found for the new unit cell. Sometimes a match indicates an isomorphous compound with very similar unit cell but different chemical elements. One example was a match between the newly collected C_44_H_54_Br_4_Zn_4_N_2_Se_6_ with a primitive cubic unit cell 17.79, 17.79, 17.79, 90.00, 90.00, 90.00 and the data base compound C_44_H_54_Br_4_Cd_4_N_2_S_6_ with unit cell parameters 17.869, 17.869, 17.869, 90.00, 90.00, 90.00, published earlier by Dean et al. at the University of Western Ontario [8]. Another example was a badly split crystal of a vanadium compound. In spite of the poor crystal quality, sufficient reflections were found to determine the unit cell and search NIST Crystal Data to match vanadyl hydrogen phosphate [9,10].

**Fig. 5:**
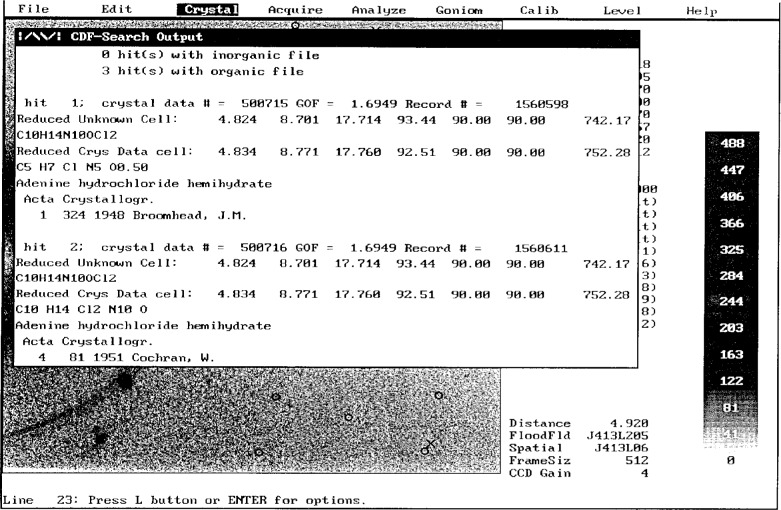
CDF search results on adenine hydrochloride hemihydrate.

Students at the Pittsburgh Summer School in crystallography are encouraged to search NIST Crystal Data prior to collecting data. An example for adenine hydrochloride hemihydrate, brought by N. Sparks [11] as a known compound, is shown in Fig. 5. Three hits in the organic file showed that the structure had been published previously three times, first in 1948.

## 4. Data Base Accessibility to Vendors and Users

The NIST Crystal Data is the only crystallographic database for which the search algorithms have been integrated with Siemens’ diffractometer control software. The volume of new small-molecule crystal structures created a real need to maximize productivity of both the researcher and the instrument. NIST Crystal Data database contains the unit-cell information needed prior to data collection. However, it was not until NIST made the database available on a simple medium, a CD-ROM in PC format, that it became feasible to access it from vendor software. It was also important that regular updates (annually) were easy to obtain through ICDD [1], and that the reasonable license fee to industrial as well as academic users made it commercially feasible.

### 4.1 Enhanced Accessibility of Crystallographic Databases

For crystallographic databases in general, some short-term recommendations can be made for today’s computing environment. At present Siemens small-molecule users prefer access on PC computers, followed by Silicon Graphics work stations. Siemens protein-crystallography users prefer Silicon Graphics workstations. CD-ROM as a media for local database storage is universal, simple, and inexpensive. While access over the Internet is attractive and growing rapidly, not every laboratory is attached. The cost of license fees, especially if access to several databases is needed, can be a barrier, particularly for industrial users. Database vendors might project maximum revenue using different scenarios ranging from low market price for a large number of users, to high market price for a smaller number of users. Market awareness of some of the databases should be enhanced, as the biggest barrier to using them is potential users lack of knowledge of what they can be used for, or indeed of their very existence.

Longer term recommendations can be more sweeping. Even now, the majority of single-crystal structures are not accessible in the databases because they have not yet been published. The barriers to publication include lack of time to prepare the publication; lack of desire to publish because the structure is not “good enough” but the chemistry is adequately determined; or the compound is proprietary. To address this, worldwide electronic deposition of unpublished data, with validation safeguards, must be strongly encouraged. Easy ways to create and search local databases of proprietary compounds could be provided. With the advent of two-dimensional detectors, the number of new single-crystal structures will grow exponentially. In the field of protein crystallography, this has already happened with the introduction of area detectors over a decade ago. In small molecule crystallography, we are just beginning the transition. From Fig. 2 we can see that if a serial diffractometer takes from a few hours to a few days to collect data, then a sustained productivity of up to 150 new structures a year is possible for one such instrument. With the new CCD diffractometer, from Fig. 4 we can see that this number could increase to as many as two to three structures per day if suitable crystals are available. Database providers will need to discover innovative ways to find and validate this data, including that in foreign-language and lesser known publications, and to update the databases ever more rapidly.

## Figures and Tables

**Table 1:** Frequency of known compounds identified by a NIST Crystal Data search at Pittsburgh Summer School in Crystallography

Year	No. structures solved	No. known compounds	
		expected	unexpected
1992	20	2	1
1993	18	0	1
1994	27	0	1
1995	19	3	2

## Footnotes

1 Certain commercial equipment, instruments or materials are identified in this paper to foster understanding. Such identification does not imply recommendation or endorsement by the National Institute of Standards and Technology, nor does it imply that the materials or equipment identified are necessarily the best available for the purpose.
